# Compassion fatigue in pediatric nephrology—The cost of caring

**DOI:** 10.3389/fped.2022.977835

**Published:** 2022-09-06

**Authors:** Aviva M. Goldberg

**Affiliations:** Section of Pediatric Nephrology, Department of Pediatrics and Child Health, Max Rady College of Medicine, University of Manitoba, Winnipeg, MB, Canada

**Keywords:** compassion fatigue, pediatric nephrology, burnout, workforce, wellbeing

## Abstract

Compassion fatigue is the result of repeated vicarious trauma from caring for those who have suffered. Although not well-researched in pediatric nephrology to date, there is reason to believe that it is a real and sustained threat to the pediatric nephrology workforce. Interventions aimed at individuals, the profession, and the organizations in which pediatric nephrologists work can create spaces to discuss and ameliorate compassion fatigue. This will result in better care for patients, more stable pediatric nephrology divisions and a stronger, more resilient pediatric nephrology workforce.

Pediatric nephrology is a rewarding field, but the work of caring for children affected by kidney disease can be often difficult and too frequently heartbreaking. Pediatric nephrologists (PNs) often diagnose diseases that portend a lifetime of medical intervention and have few cures. We often meet children and their families in emergency or intensive care settings, where we need to discuss devastating, life altering illnesses and make rapid decisions about complicated and potentially morbid treatments. While the profession can provide immense personal and professional satisfaction, the emotional toll of the work can be heavy, especially when compounded over many cases and many years.

While the field is rightly paying increasing attention to the emotional and mental health impact of pediatric kidney disease on children and their families, there has been less focus on the impact of these situations on health care workers. As Figley noted when first characterizing compassion fatigue- “nearly all of the attention has been directed to people in harm's way, and little to those who care for and worry about them” (1). Compassion fatigue is a real threat to wellness within the pediatric nephrology profession and to recruiting and retaining a healthy pediatric nephrology workforce. This article will outline what compassion fatigue is, what little is known about in pediatric nephrology and how it can be addressed by individuals, the profession, and the institutions in which we work.

## What is compassion fatigue? How is it measured?

Compassion fatigue (CF) was first described in the nursing and psychotherapy literature and is more recently being studied and discussed amongst the physician community.As described in an interview of emergency nurses early in the characterization of the syndrome, compassion fatigue was recognized as a “unique” form of burnout that affected those in the “caring professions” (2). Those interviewed recognized that professional caregivers needed to be constantly emotionally available and that this emotional work could take them away from their own wellness. It was noted that caregiving professionals also work in systems that expect more and more of individual employees and make decisions without the health and wellness of staff top of mind. Facing repeated stresses in a system that does not address them adequately gave those experiencing CF little room for individual or group support. Though much has been studied and written about compassion fatigue since the early 1990 s, many of these same themes will seem familiar to the modern pediatric nephrologist.

Compassion fatigue is now understood as a type of secondary or vicarious stress due to the repeated and ongoing exposure to the suffering of others (1). Those in the helping professions, be they physicians, social workers, or others, are repeatedly traumatized by witnessing the suffering of their patients and clients (3). This traumatization can lead to a decreased capacity to empathize with future patients (4). CF is closely related to burnout, defined as chronic occupational stress characterized by emotional exhaustion, feelings of depersonalization and career dissatisfaction (5, 6). While the two are interrelated, CF can be conceptualized more as the stress that originates in the responsibility to care for victims of trauma, while burnout originates in workplace factors like overwork and under-appreciation. As well, burnout is a gradual wearing down process, whereas compassion fatigue can sometimes be more immediate and specific, triggered by an individual event (7). Part of addressing CF is the cultivation of its “antidote”- compassion satisfaction (CS). CS is what many of us will remember as one of the reasons we chose to go into medicine- the sense of fulfillment derived from caring for others (8).

CF is a problem for patients because it can impede the ability of health care workers to bring their best to their patients. It is a problem for health systems because workers with CF may have greater sick calls, or consider leaving the profession (9, 10). It is, of course, a problem for those with CF themselves because it can result in job dissatisfaction, cognitive and even physical distress similar to that of post-traumatic stress disorder, leading to feelings of hopelessness and helplessness (11).

CF can be measured by several validated scales. The Professional Quality of Life Scale, the Secondary Traumatic Stress Scale, the Impact of Event Scale- Revised and others have all been used as measures of CF in published studies (11–14). The modified Compassion Fatigue and Satisfaction Self-Test for Helpers has been used in a number of cross sectional pilot studies of other pediatric subspecialties in recent publications (15–21). It is easily searchable online for self test versions. These tests explore symptoms of compassion fatigue across three domains:

1. Psychological (e.g., intrusive thoughts, feeling numb, avoiding the patient or situation).
2. Cognitive (e.g., increased cynicism, increased personal vulnerability).
3. Interpersonal (e.g., withdrawal from the larger treatment team, difficulty trusting others) (7).

## What do we know about compassion fatigue in pediatric nephrology?

While compassion fatigue has not yet been well-studied in PNs, there is evidence to suggest that it is a risk for this workforce. When Figley first characterized compassion fatigue amongst trauma workers, he posited that there were 4 factors that led to its development (1).

1. Empathy is a major resource for trauma workers to help the traumatized.
2. Most trauma workers have experienced some traumatic event in their lives.
3. Unresolved trauma of the worker will be activated by reports of similar trauma in clients.
4. Children's trauma is provocative for therapists.

Many pediatric nephrologists will easily identify these four factors in the work that we do. All our work deals with children and empathy is an essential tool in our toolbox. Our patients do experience trauma related to their kidney diseases and diagnoses, often both physical and psychological. Some of this trauma even occurs as a result of necessary treatment decisions for which we are responsible (e.g., surgeries for dialysis catheter placement). Pediatric nephrologists are therefore, in a very real sense, trauma workers. Children with kidney disease often need high technology interventions like dialysis and transplant, which are time consuming and complicated and require a high cognitive load from the medical provider (6). The diseases we work with are often exceedingly rare, making it difficult to protocolize some aspects of care and provide peer support or education to families. Since kidney disease can be relatively asymptomatic until it is severe, the diagnosis of end stage disease often comes at families as a shock, leading to difficult and emotionally charged conversations at the very same time when big decisions are required. An article about nephrology nurses identified that the relationship of renal care teams to their patients can increase the risk for compassion fatigue for a number of reasons. Nephrology care, especially hemodialysis, requires frequent and intense contact between providers and patients, often multiple times a week for years on end (22). Even when children can receive a kidney transplant and move out of the hemodialysis unit, they need near constant monitoring and frequent follow-up visits. While this can lead to extraordinarily strong and positive relationships between patients and their providers, negative outcomes have high impact.

In addition to the intrinsic factors of caring for children with kidney disease, the systems in which we work can contribute to both compassion fatigue and burnout. The burdens of electronic medical record keeping and interactions with health insurers can take physicians away from direct patient encounters and contribute to burnout and other issues (23). Healthcare austerity and the rapidly shrinking pediatric nephrology workforce have all contributed to a do-more-with-less message, low morale, and the sense of being uncared for by the organizations in which many work (24). This lack of institutional support can lead to poor wellbeing in multiple domains, and make it difficult to find the time for self-care.

There appear to be no published studies specifically examining CF in PNs but there have been other studies of other pediatric subspecialists. Nursing studies in pediatric oncology, emergency medicine, palliative care and the care of medically complex children identify CF as a common issue (25–28). Recent studies have examined CF in pediatric emergency medicine physicians, pediatric palliative care providers, neonatologists, pediatric hematologists oncologists and pediatric surgeons (15, 16, 18, 19, 21). CF rates in these physician groups ranged from 16 to 22 percent of respondents, with female sex, burnout score, distress about a clinical situation and teaching burden associated with higher scores.

There is some evidence that PNs actually fare better in some measures of wellbeing than do our adult colleagues or those in other pediatric subspecialties. The Sustainable Pediatric Nephrology Workforce Project (SUPERPOWER) recently published their findings on burnout among US pediatric nephrologists survey done in February–April 2020 (24). Perhaps surprisingly, PNs reported relatively low rates of burnout- 13% among PN fellows and 16% among PN faculty. This rate is markedly lower than burnout studies in most other physicians groups, where rates as high as 70% have been reported (29). A study of Polish pediatric nephrologists conducted just prior to pandemic found somewhat higher rates of burnout, with 26.8% reporting at least medium level of burnout in all three dimensions and 8.2% with high three-dimensional burnout (30). Burnout correlated to other markers of poor wellbeing, including lower resilience, lower self-compassion, lower perceived quality of life, and higher stress (25). So, while not specifically studied in this group, it is reasonable to assume that there are some pediatric nephrologists who have personal and workplace factors that place them at risk for CF, and others who are faring well-despite the challenges of the work.

The COVID-19 pandemic has, of course, increased some of the risks for compassion fatigue. A survey of non-nephrology pediatric subspecialists in the early days of the pandemic found that compassion fatigue scores did not differ significantly overall between pre- and early pandemic samples (17). However, for those with CF, distress about mental health and future uncertainty were associated with higher scores. As well, the technology that helped us continue to provide care throughout the pandemic may contribute to CF. Making “real” connections with patients is one of the ways in which healthcare workers can protect themselves from CF. A recent study of telehealth visits in pediatric nephrology found that while the technology is beneficial in many ways, it can make it more difficult to create personal connections to patients when in virtual spaces (31). Despite the undeniable challenges of the pandemic, there is also evidence of resiliency. A study of Spanish healthcare workers in the first phases of the pandemic found that while CF and burnout were concerns during the pandemic, compassion satisfaction increased due to the ability to help others in a disaster situation (32). Cultivating compassion for self and others has been associated with lower rates of pandemic related depression and higher sense of social safeness (33).

## Addressing CF in the pediatric nephrology workforce- What can be done?

While it is important for pediatric nephrologists to pay attention to their own wellbeing and identify when they might be prone to CF, self-management strategies cannot be the only method to address the issue. Rourke has suggested that compassion fatigue must be addressed at three levels: personal, professional, and institutional (7). Following this framework, I suggest a few interventions at all three of these levels that have some evidence for success from other professional groups, and examples of how this could be enacted in a pediatric nephrology practice and the evidence for their success from other professional groups.

A. **Individual strategies and interventions**

1. **Enhance compassion satisfaction** (8)- CS can be cultivated through deliberate attention to interactions we have with patients. For example, a study of oncology nurses found that those who were seen as compassion exemplars spoke of three types of moment-making as key to their resiliency (34).

a. Moments of connection- connecting to patients and families through deliberate attention to their experience (e.g., spending some time in transplant clinic asking about a patient's siblings, hobbies, or upcoming family milestones).
b. Making moments matter- participating in activities to enhance the connection between patients and their caregivers (e.g., attending the hemodialysis patient holiday party, volunteering at Kidney Camp).
c. Energizing moments- bringing energy and a positive attitude, even when the work can be difficult (e.g., deliberately starting each day with a moment of gratitude and commitment to the work that lies ahead).

2. **Participate in workplace based interventions**- There are many individual and group activities that can contribute to psychological wellbeing. Many of these have been trialed with success in those experiencing CF. Mindfulness based stress reduction and other techniques have been used to reduce CF in other healthcare providers (35, 36). A study of pediatric oncology nurses employed multi-modal support including education on CF and CS, nutritional and fitness support, tangible methods for grief and remembrance and the creation of a respite room (3). Pre and post intervention testing showed that compassion fatigue significantly decreased 4 months after the intervention.
3. **Engage in self-care beyond the workplace**- This is, admittedly, easier said than done. Many busy PNs are busy with clinical care, administration, teaching, and research responsibilities, and have family commitments that can impact personal time (37). However, it is essential that PNs find ways to “fill their own buckets” by attention to rest, nutrition, physical activity, and enjoyable activities outside of the workplace (20, 26). It would be irresponsible to suggest that physicians should heal themselves, however, unless the profession and institutions create space for this care to occur. Therefore, prodessional and institutional level interventions are also required.

B. **Professional strategies and interventions**
  As pediatric nephrologists, we have a duty to our colleagues and our trainees to better understand and address CF. As with other recent work of the ASPN (American Society of Pediatric Nephrology) workforce committee to examine burnout, remuneration and other issues, attention to CF can help lead to a stronger PN workforce. Some suggestions for the profession to better recognize and ameliorate CF include:

1. **Study the problem**- Much of the CF literature to date has been in non-physician groups, in oncology, palliative care and emergency medicine. While there is much to learn from the experiences of our colleagues, it is important to understand how CF and CS are expressed in the PN workforce. Finding the risk and resiliency factors unique to our profession can help better prepare and intervene when CF becomes an issue for our colleagues.
2. **Give permission to share**- Particularly during the pandemic, healthcare providers may express a tendency to minimize our perceptions of our own stress and suffering when compared to those of our patients. Most of us are much healthier than most of our patients, in a better socioeconomic stratum, and benefit from the prestige and remuneration of our medical careers. It can therefore feel self-centered to acknowledge or discuss our own compassion fatigue or other psychological threats to our wellbeing. While admirable to put the needs of patients first, this lack of communication can lead to a mutual pretense in which many are thinking and suffering in similar ways, but no one is comfortable speaking aloud (7). Venues to discuss and intervene on CF, like academic rounds and professional development activities, should be cultivated by the profession. As an example, a recent article by the editor-in-chief of a prestigious adolescent medicine journal models how leaders who display vulnerability can inspire others to seek help and support each other (38).
3. **Fulfill the “duty to warn”**- Trainees entering pediatric nephrology should be warned that compassion fatigue is a risk to PN care, but that compassion satisfaction is one of the many benefits (1). Self-care, boundary setting and resilience building should be components of pediatric nephrology fellowship training. Ideally this work builds on tools that have been incorporated into earlier medical school and residency training. For practicing faculty, many of who may not have had such training themselves, continuing professional development opportunities should be available.
4. **Make space for meaningful work**- Returning to the reasons pediatric nephrologists chose to enter the field can be an important factor in cultivating compassion satisfaction. A 2010 survey of pediatric nephrology fellows found that engaging with renal physiology, the academic setting, critical care nephrology, and outpatient nephrology were attractive features to the majority of PN trainees (39). Nephrology divisions can encourage such job satisfaction by allowing pediatric nephrologists to diversify their clinical work and pursue teaching and research opportunities that they find satisfying and rewarding.
5. **Group support and debrief-** Talking about traumatic experiences with trusted colleagues, as through critical incident debriefing sessions, can help to manage the posttraumatic effects and assure PNs that they are not alone in having complicated feelings around these issues (40, 41). As with many types of stress, grief can be lessened when it is shared (15). Likewise, celebrating group and individual successes in clinical and academic life can enhance work engagement and compassion satisfaction cultivated in shared moments of celebration and encouragement.
6. **Advocacy and equity**- Acknowledging and addressing CF as a professional can do much to educate our colleagues and encourage them to seek help when needed. While much of the research in CF has been done in relatively well-resourced countries, we should also be mindful of our roles in the international community. A study of the global nephrology workforce found a 90-fold difference in nephrologist density between the highest and lowest income countries (42). Workload factors and the inability to provide care to all who need it may contribute to feelings of helplessness and to CF (43). Understanding and addressing these disparities in global access should be a priority for the profession in order to provide better care for children with kidney disease but also to better support our colleagues working in these challenging environments.

C. **Institutional factors**
  While interventions for CF need to be a priority for individuals and the profession they should also be a priority for the institutions in which we work and collaborate.

1. **Work engagement**- In a group of Chinese hemodialysis nurses, work engagement was identified as one of the mediating factors reducing compassion fatigue (9). Work Engagement can be enhanced by institutions that recognize and reward the work of employees, provide venues to hear and understand concerns and show action on the issues that matter to staff. Healthy work environment factors that have been shown it increase compassion satisfaction are authentic leadership and meaningful recognition (44). It is therefore important that this engagement be genuine and sustained. Acts of recognition that are seen as performative or minimal can easily backfire (e.g., giving staff discounts on yoga classes without listening to the distress that they are experiencing in the workplace) (45).
2. **Faculty support services -** It can be difficult to ask for help when one is experiencing CF or other psychological distress, so institutions and those in positions of leadership should create sources of support that are easily accessible. Similar to the offices of Student Affairs that are available to medical students and residents, programs that allow faculty access to personal counseling, mental health supports, cognitive behavioral therapy and even group wellbeing events can help lower the stigma around asking for help. Employee benefits should include access to psychological support and counseling when needed, and discretionary plans (like healthcare spending accounts) should include consideration for wellness based services like relaxation massage.
3. **Workload and staffing-** A recent article by Weidemann and colleagues identified that understaffing and poor remuneration were significant threats to the wellbeing of pediatric nephrologists (37). A 2015 study of American pediatric nephrologists found 47% of respondents believed that their division staffing to be insufficient (46). Thirty three percent of respondents reported a plan to reduce clinical activities and of those (other than those planning to retire) dissatisfaction with work life balance (45%) was as the most frequently reported reason. In the same study, respondents who said that they were not sure that they would choose the profession again (about 1/3 of the total replies) cited workload, work-life balance, financial factors, and institutional support among their reasons. These results call for better staffing and remuneration in the PN workforce. Not only does adequate staffing allow for safe patient care, but it allows people to engage in the very activities that are key to CF management. Institutions should commit to better workload management and recruitment and retention strategies that ensure adequate staffing.
4. **Sustained attention**- Individual interventions will not be enough to combat CF nor is it enough to intervene once and expect sustained results. For example, while the previously referenced study in pediatric oncology nurses showed some benefits to the intervention at 4 months, the results were not sustained, with CF scores returning to baseline within 6 months of the intervention. Therefore, institutions which seek to reduce CF among their faculty and employees should make intentional and sustained efforts to create and improve working conditions in ways that enhance wellness.

## Conclusions

One of the first steps in combatting compassion fatigue is to measure it effectively within our field. We need to measure and report on CF, burnout, and compassion satisfaction in ways similar to our nursing colleagues and other pediatric subspecialties. We need to ask our colleagues and ourselves what we love about our work and what needs to change. Personal resiliency factors should be nurtured. The profession should advocate for CF education and resources in both pediatric nephrology training programs and continuing professional development as well. Finally, institutions should be attentive to CF in the PN workforce and address it. We need to hold the systems in which we work accountable not only for patient outcomes but for the wellbeing of staff and faculty. For those of us working in resource rich environments, we also need to recognize the areas in which we have privilege, and include the perspectives and needs of the global pediatric nephrology workforce in our advocacy efforts. Doing this “self-work” is not selfish; we are better physicians to our patients when we are better to ourselves. Pediatric nephrology will always be a profession where we walk along difficult journeys with our patients. There will be trauma and times that will test us. However, we can shape our own resiliency factors and the environments in which we work to be the most supportive as possible. Compassion is a necessary component to excellent pediatric nephrology care, but compassion fatigue is both avoidable and treatable.

## Author contributions

Author AG conceived the concept and wrote the manuscript.

## Conflict of interest

The author declares that the research was conducted in the absence of any commercial or financial relationships that could be construed as a potential conflict of interest.

## Publisher's note

All claims expressed in this article are solely those of the authors and do not necessarily represent those of their affiliated organizations, or those of the publisher, the editors and the reviewers. Any product that may be evaluated in this article, or claim that may be made by its manufacturer, is not guaranteed or endorsed by the publisher.
